# Polarization Induced Deterioration of Reinforced Concrete with CFRP Anode

**DOI:** 10.3390/ma8074316

**Published:** 2015-07-15

**Authors:** Ji-Hua Zhu, Liangliang Wei, Miaochang Zhu, Hongfang Sun, Luping Tang, Feng Xing

**Affiliations:** 1 Guangdong Province Key Laboratory of Durability for Marine Civil Engineering, School of Civil Engineering, Shenzhen University, Shenzhen 518060, Guangdong, China; E-Mails: zhujh@szu.edu.cn (J.-H.Z.); liangwei.szu@gmail.com (L.W.); szuzhumc@gmail.com (M.Z.); 2 Department of Civil and Environmental Engineering, Chalmers University of Technology, Gothenburg SE-412 96, Sweden; E-Mail: tang.luping@chalmers.se

**Keywords:** polarization, interface deterioration, reinforced concrete, CFRP anode

## Abstract

This paper investigates the deterioration of reinforced concrete with carbon fiber reinforced polymer (CFRP) anode after polarization. The steel in the concrete was first subjected to accelerated corrosion to various extents. Then, a polarization test was performed with the external attached CFRP as the anode and the steel reinforcement as the cathode. Carbon fiber reinforced mortar and conductive carbon paste as contact materials were used to adhere the CFRP anode to the concrete. Two current densities of 1244 and 2488 mA/m^2^, corresponding to the steel reinforcements were applied for 25 days. Electrochemical parameters were monitored during the test period. The deterioration mechanism that occurred at the CFRP/contact material interface was investigated by scanning electron microscopy (SEM) and X-ray diffraction (XRD) techniques. The increase of feeding voltage and the failure of bonding was observed during polarization process, which might have resulted from the deterioration of the interface between the contact material and CFRP. The formation and accumulation of NaCl crystals at the contact material/CFRP interface were inferred to be the main causes of the failure at the interface.

## 1. Introduction

Reinforced concrete structures may suffer from premature failure induced by the corrosion of the reinforcing steel embedded in the concrete, which implies that a huge investment in strengthening, repair, and rehabilitation of the degraded structures in order to reach their targeted service lives. Chloride ingress is one of the major causes of steel corrosion, which can lead to concrete cracking due to the expansion exerted by corrosion products [1,2]. The corrosion of steel by chloride in concrete is generally understood as an electrochemical process [3]. Nowadays, various well-developed methods are available for determination of the chloride diffusivity [4] and steel corrosion control [5]. Among them, impressed current cathodic protection (ICCP) is considered one of the most effective methods [6,7] and usually provides sufficient protection [8]. In an ICCP system, a cathodic current is applied to the reinforcing steel, resulting in the shifting of the steel potential towards a level at where the corrosion rate is negligible [6,7].

It is of crucial importance to select a suitable anode for the delivery of the protection current from the surface through the concrete to the steel rebar. Different types of anode materials have been developed, including thermal-sprayed zinc anodes [9], thermal-sprayed titanium anodes [10,11], titanium mesh [12] and conductive paint or overlay coating anodes [13,14]. Industry is still looking for new anode materials to satisfy the requirements of bond efficiency, installation convenience, and lower cost.

Carbon fiber reinforced polymer (CFRP) is generally considered as a lightweight material with excellent mechanical strength and electrical conductivity [15]. Because of these advantages, CFRP has been increasingly used in civil engineering, especially in structural strengthening of concrete structures. However, the remarkable electrical conductivity of CFRP was less recognized in civil engineering. It is reported that CFRP can be used as the anode material of ICCP system in electrochemical repairing of reinforced concrete structures. Gadve *et al*. [16,17] reported the test results of reinforced concrete structures lollipop specimens and beams with CFRP serving as an impressed current anode. Van Nguyen *et al*. [18] studied the performance of CFRP fabric and rods as impressed current anodes in calcium solution and concrete. Consequently, the idea of combining the CFRP’s dual functional characteristics of both mechanical properties and electrical conductivity was pointed out which may lead to a novel method of renovating structures. The reported results [19,20] compared the ultimate strengths of specimens with CFRP performing dual roles (both strengthening and anode material) against control specimens in which CFRP was only used for structural strengthening.

The above studies mainly focused on the efficiency of using CFRP anode in ICCP against steel corrosion, whereas degradation of the system is also critical, especially for the long-term performance in the service life period. The degradation depends on various aspects including the behavior of anode and contact (adhesive) materials, as well as the interface behavior of both anode/contact material and concrete/contact material. Recently, Zhu *et al*. [21] investigated the behavior of CFRP anode in the simulated ICCP system. No significant degradation in electrical and mechanical properties was found of CFRP subjected to anodic polarization at the selected applied current densities, which demonstrated that CFRP could be successfully used to simultaneously carry out the dual functions of impressed current anode and structural strengthening.

In the present paper, a great interest has been developed in studying the deterioration of ICCP system of reinforced concrete with CFRP anode. Polarization tests with large current densities were adopted to accelerate possible deterioration phenomena close to the CFRP anode. Influence of initial corrosion levels and different contact materials, as well as polarization extents were studied.

## 2. Experimental Program

The following experimental program was carried out in this study.

External current was impressed to induce the corrosion of steels embedded in concrete specimens. Polarization was then applied to the corroded specimens with CFRP anode and monitoring the electrochemical parameter (feeding voltage). Microstructural changes at the CFRP/contact material interface were examined after polarization process.

### 2.1. Specimen Preparation

**Figure 1:**
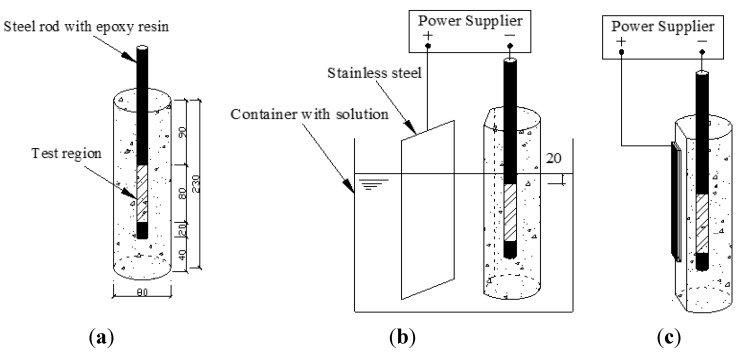
Schematic view of test setup (**a**) cylindrical concrete specimens; (**b**) corrosion acceleration; (**c**) polarization system of concrete specimens with CFRP anode. (Unit: mm)

Eighteen reinforced concrete cylinders were prepared, each with a diameter of 80 mm and height of 230 mm, as shown in Figure 1a. The concrete was mixed using 456 kg/m^3^ of Portland cement (Conch 32.5, Anhui Conch Cement CO. LTD, Wuhu, China). The water to cement ratio was 0.45 and the sand ratio was 0.37. The coarse aggregate was crushed stone with a maximum size of 30 mm. The fresh concrete was cured in a chamber at a temperature of 25 °C and relative humidity of 95% for 28 days after casting. Ribbed steel (HRB335, ShaoguanIron CO. LTD, Shaoyang, China) with a diameter of 16 mm was placed axially in the center of the cylindrical concrete specimens. A hole of 1.5 mm in diameter and 10 mm in length was drilled at the end of each steel rod to allow electrical connection with an external wire. An 80 mm segment of each steel rod was exposed for testing and the rest was sealed by epoxy resin for isolating. Thus, the surface area of steel participating in polarization process was 4020 mm^2^. After 28 days of curing, the cylinders were cut to expose a planar surface for the adhesion of CFRP anode.

### 2.2. Corrosion Acceleration Process

Accelerated corrosion of the steel was applied prior to the polarization process. The impressed current method was used to introduce accelerated corrosion to the in-concrete steel, which played the role of anode while a stainless steel plate was used as the cathode Figure 1b. The specimens were immersed in 3.5% NaCl solution during the accelerated corrosion process with the solution level maintained 20 mm above the exposed part of the ribbed steel, as shown in Figure 1b. A constant current of 25 mA per specimen was impressed for 24 h, 48 h, and 96 h to introduce three corrosion levels to the total of 15 specimens (Table 1). The voltage between anode and cathode was monitored every 20 minutes with a data logger.

**Table 1:** Parameters used for corrosion acceleration and polarization test.

Groups	Specimens	Corrosion acceleration time (h)	Contact material	Polarization current (mA)	Polarization current density (mA/m^2^)
C_24_	C_24_I_0_M	24	mortar	0	0
	C_24_I_0_P	24	paste	0	0
	C_24_I_5_M	24	mortar	5	1244
	C_24_I_5_P	24	paste	5	1244
	C_24_I_10_M	24	mortar	10	2488
	C_24_I_10_P	24	paste	10	2488
C_48_	C_48_I_0_M	48	mortar	0	0
	C_48_I_0_P	48	paste	0	0
	C_48_I_5_M	48	mortar	5	1244
	C_48_I_5_P	48	paste	5	1244
	C_48_I_10_M	48	mortar	10	2488
	C_48_I_10_P	48	paste	10	2488
C_96_	C_96_I_0_M	96	mortar	0	0
	C_96_I_0_P	96	paste	0	0
	C_96_I_5_M	96	mortar	5	1244
	C_96_I_5_P	96	paste	5	1244
	C_96_I_10_M	96	mortar	10	2488
	C_96_I_10_P	96	paste	10	2488

### 2.3. Polarization Process

Schematic view of the polarization system with CFRP anode is shown in Figure 1c. CFRP was adhered to the planar surfaces of the concrete specimens by contact materials.

Commercial CFRP strips were used in this study, since this kind of material maintained good performance as anode in previous studies [21]. According to the information given by the manufacturer, CFRP strips are made of multi-layer carbon fibers bounded by LAM-125/LAM-226 laminating epoxy (Pro-Set Inc., Bay City, MI, USA). Each layer consists of weft-warp-knit carbon fibers combined by the epoxy. The carbon fiber used in CFRP was Toray T700 with volume fraction of 60%. The CFRP plates were machined to 25 × 150 mm^2^ to be compatible with the planar surfaces of the specimens, as shown in Figure 1c.

Two kinds of contact materials were used to adhere CFRP to the concrete specimens. One is a made-in-laboratory carbon fiber reinforced mortar (abbreviated to mortar in this paper) and the other is a commercial conductive carbon aluminosilicate paste (abbreviated to paste in this paper). The mortar was prepared by using carbon fibers of length 3–5 mm in the amount of 1.5% of cement by mass, fine river sand, and cement with a water to cement ratio of 0.45 and cement to sand ratio of 1.0. Methylcellulose in the amount of 0.4% of cement by mass was first added to water as a dispersing agent to disperse carbon fibers uniformly before adding cement and fine sand. The commercial conductive carbon-aluminosilicate paste has a good conductivity due to the graphite particles, with a volume resistivity of 0.01–0.001 Ohm·cm.

The polarization test started after 7 days of adhering CFRP to the concrete to allow hydration of the cement. Two constant currents, *i.e.*, 5 and 10 mA, were applied corresponding to polarization current densities of 1244 mA/m^2^ and 2488 mA/m^2^, respectively, relative to the surface area of steel. The test duration was 25 days for all specimens. The feeding voltage between the steel and the CFRP was periodically measured with a high impedance multimeter during the test.

The 18 specimens were divided into three groups and labeled according to the corrosion level, polarization current, and types of contact material, as shown in Table 1. The label C_24_I_5_M, for example, defines the specimen with a corrosion time of 24 h (C_24_), polarization current of 5 mA (I_5_), and carbon reinforced mortar (M) as the contact material. The conductive carbon-aluminosilicate paste is indicated by P in the labels. It should be noted that the specimens with I_0_ are the control specimens without polarization, which were prepared for comparison purposes.

### 2.4. Microstructure Analysis

Microstructure observations were performed by Digital Camera, Scanning Electron Microscope (SEM), and X-ray Diffraction (XRD). XRD measurements were conducted on a Bruker D8 advance instrument with a CuKα source at 40 kV and 200 mA. The instrument has built-in phase identifying software to analyze the XRD spectra. SEM was performed on a Hitachi S-3400N scanning electron microscope with accelerating voltage of 15 kV and working distance of approximately 10 mm. Before loading the specimen into the SEM chamber, the specimens were sputter-coated with gold to form a conductive surface. The corrosion depth was assessed to reflect the influence of the polarization process on CFRP, according to which the corrosion rate (corrosion depth/duration time) was also estimated. The corrosion depth was measured by removing the corroded part of CFRP with a knife and then measuring the thickness of the remaining un-corroded part. The corrosion depth was then calculated by subtracting the un-corroded thickness by the initial thickness.

## 3. Results and Discussion

### 3.1. Accelerated Corrosion of Steel

Corrosion was introduced to the steel imbedded in the concrete specimens using an accelerated corrosion method so that, when polarization was applied, the steel had already suffered from corrosion to simulate real-life conditions.

**Figure 2:**
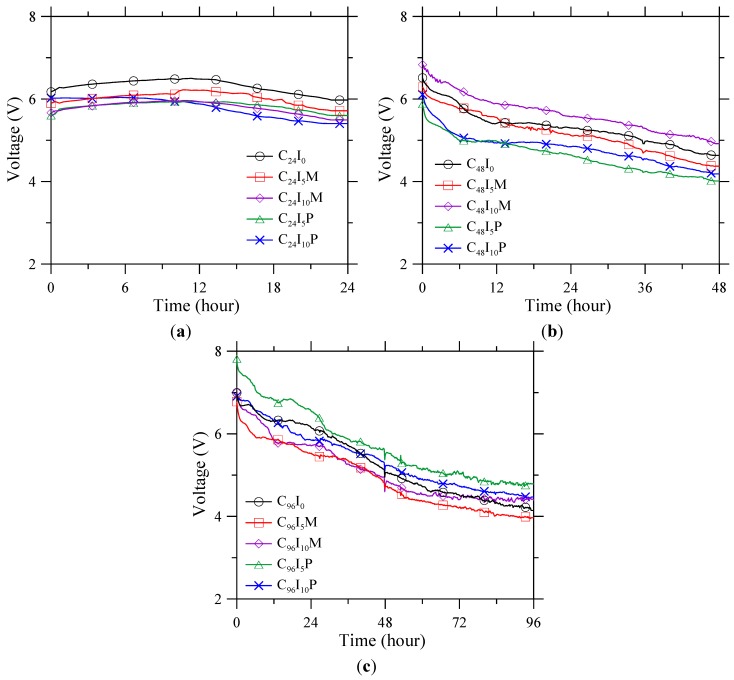
Measured voltage between ribbed steel anode in concrete and stainless steel cathode in solution during the periods of accelerated corrosion for (**a**) 24 h; (**b**) 48 h; and (**c**) 96 h.

The measured voltage between the ribbed steel anode and the stainless steel cathode is presented in Figure 2. The voltage of group C_24_ shows no significant change during the corrosion acceleration process, with the voltage distributed over the range 5–6 V. Meanwhile, the voltage of groups C_48_ and C_96_ decreased with time, from 6 to 8 V at the beginning down to 4–5 V at the end. The voltage drop indicates the decrease of resistance between the anode and cathode.

### 3.2. Feeding Voltage during Polarization

**Figure 3:**
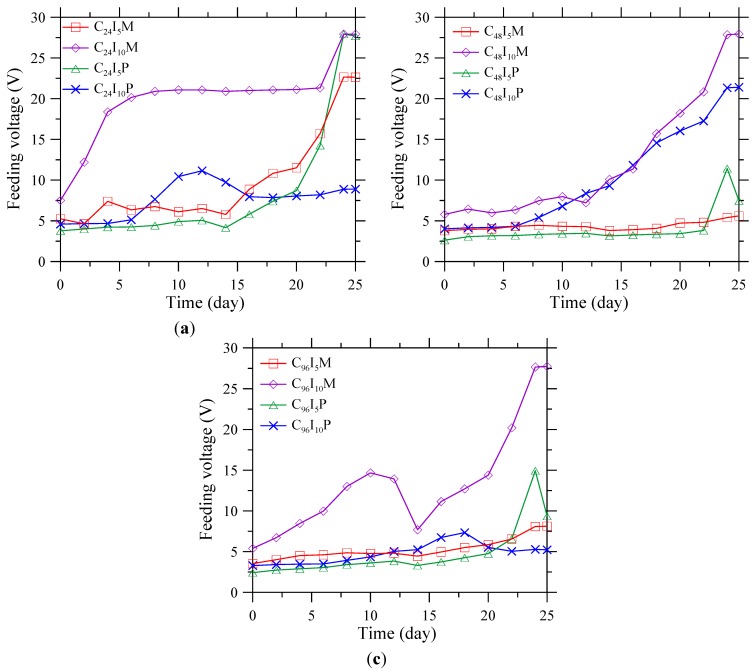
Feeding voltage between steel in concrete and CFRP for group (**a**) C_24_; (**b**) C_48_; and (**c**) C_96_ during polarization test.

The feeding voltage of the polarization system with CFRP anode was periodically monitored during polarization process. The feeding voltage curves are shown in Figure 3. The feeding voltage seems to be associated with the corrosion level, polarization current density, and the types of contact materials. For group C_24_, the feeding voltage of C_24_I_5_M and C_24_I_5_P stabilized at around 5 V during the first 15 days, and then increased to 27.5 V, which is four times more than the original voltage as shown in Figure 3a. At the polarization current of 2488 mA/m^2^, C_24_I_10_M and C_24_I_10_P behaved differently. The voltage for C_24_I_10_M showed an initial increase from 5 to 20 V in the first five days, and then remained stable over the following 15 days. C_24_I_10_P showed a peak at approximately 12 days. However, in general, the voltage showed an increasing trend. For groups C_48_ and C_96_, the results in Figure 3b,c show that, at a current density of 1244 mA/m^2^, the voltage increased very slowly from 2 to 10 V; while at 2488 mA/m^2^, the feeding voltage increased more, from 2 to 27 V.

Generally, the measured feeding voltage indicates an effective electrical connection, although the resistance increased during the test period. The increase of feeding voltage should be related to the loss of electrochemical activity of the system [22]. The underlying mechanism will be discussed in the following sections. Moreover, the specimens with current density of 1244 mA/m^2^ had more stable electrical connections than those with 2488 mA/m^2^ at the same corrosion level and with the same type of contact material.

### 3.3. Interface Examination

An increase of feeding voltage was observed during polarization process, as shown in Figure 3, indicating a degradation of electrical connection. To investigate the failure mechanism, microstructure observations were performed using digital camera, SEM, and XRD on specimens C_24_I_0_P, C_24_I_5_P, and C_24_I_10_P with carbon paste and C_24_I_0_M, C_24_I_5_M, and C_24_I_10_M with mortar.

**Figure 4:**
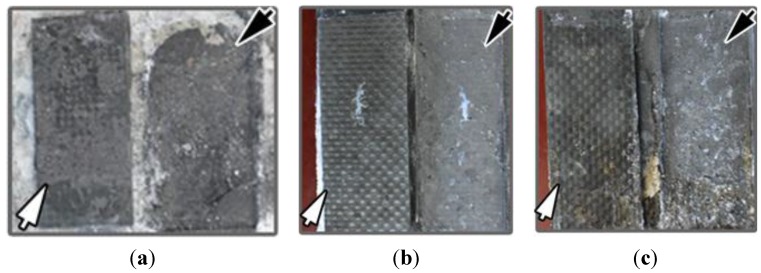
Photographs of the failure interface between CFRP and carbon paste of specimen (**a**) C_24_I_0_P; (**b**) C_24_I_5_P and (**c**) C_24_I_10_P. The black arrows indicate carbon paste still attached to the concrete; the white arrows show the CFRPs peeled from the carbon paste.

The detached interfaces are shown in Figure 4, where the failure surface of CFRP is indicated by white arrows and the failure surface of the contact materials by black arrows. Of the specimens with carbon paste as the contact material, the CFRP anode of C_24_I_0_P (no polarization current applied) seemed to have fractured from the inside of the contact material, since part of carbon paste was still attached to the CFRP surface. Specimen C_24_I_5_P, to which a 5 mA current was applied, showed a smooth failure interface between CFRP and carbon paste, indicating a reduction of adhesive bonding of the carbon paste. When the current increased to 10 mA, the failure still occurred on the interface between CFRP and carbon paste. In addition, the CFRP surface seemed more corroded.

**Figure 5:**
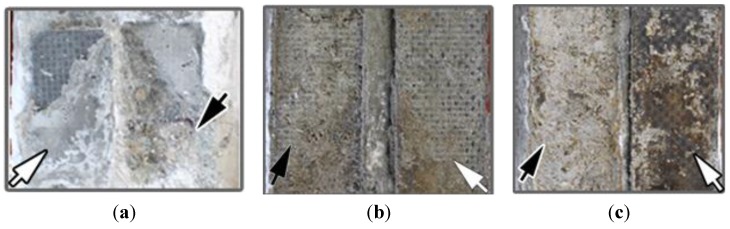
Photograph of the interface between CFRP and mortar of specimen (**a**) C_24_I_0_M; (**b**) C_24_I_5_M and (**c**) C_24_I_10_M. The black arrows indicate mortar still attached to the concrete; the white arrows show the CFRPs peeled from the mortar.

Similar failure phenomena were observed for specimens C_24_I_0_M, C_24_I_5_M, and C_24_I_10_M with mortar as the contact material (Figure 5). In these specimens the CFRP seemed more severely corroded than for the samples with carbon paste as the contact material, since mortar usually contains more pores than paste due to the use of aggregate and the existence of a transition zone, which provides additional paths for ionic transport.

In both cases (carbon paste and mortar as contact material), the failure after application of current occurred at the interface between contact material/CFRP indicating that the interface was the weakest part of the system. Thus the failure mechanism will be related to the chemical change of both contact materials and CFRP, especially the part close to the interface region. Thus the microstructure of those two parts was studied in the following sections.

### 3.4. Contact Materials

The chemical change and microstructure of the contact material at the interface were investigated by XRD and SEM techniques.

**Figure 6:**
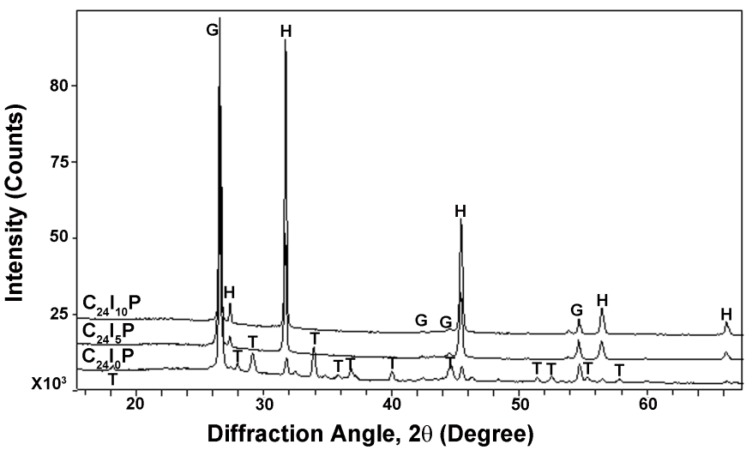
XRD spectra identification of carbon paste subject to varied current densities. G-Graphite (C), H-Halite (NaCl), T-Trona [Na_3_H(CO_3_)_2_•2H_2_O].

For the carbon paste contact material, The XRD spectra and identification of the three specimens subject to varied current with paste as contact material are shown in Figure 6. The C_24_I_0_P sample consisted of three crystalline phases: graphite (C) is the conductive constituent which provided the conductivity of the contact material; trona [Na_3_H(CO_3_)_2_•2H_2_O] is the crystalline product formed during the hardening of the contact material; and halite (NaCl) diffuses from the NaCl solution. When the current was increased from 0 to 5 mA and 10 mA, the graphite peaks showed no significant change. However, the height of the NaCl peak increased, indicating more halite (NaCl) was produced. Trona [Na_3_H(CO_3_)_2_•2H_2_O] disappeared, which might be due to the acidification effect around the anode region which converted trona to NaCl, water, and CO_2_.

**Figure 7:**
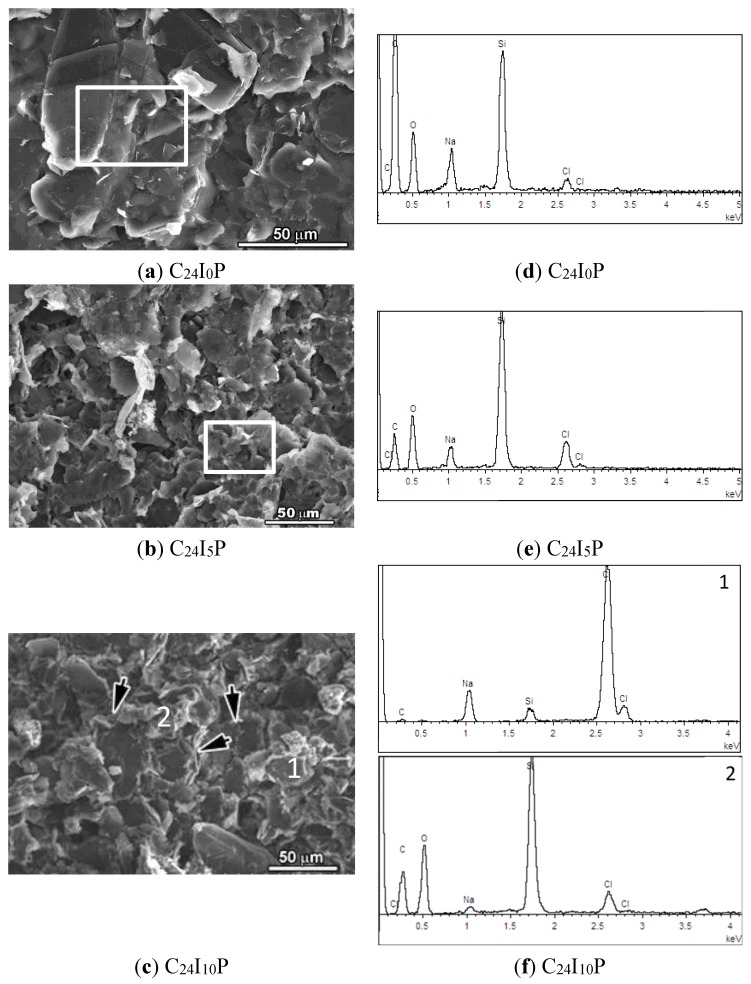
SEM images of the carbon pastes of specimen (**a**) C_24_I_0_P; (**b**) C_24_I_5_P and (**c**) C_24_I_10_P. The element analysis results (EDS) of the corresponding area indicated by rectangular frame and 1 and 2 are shown in (**d**); (**e**) and (**f**).

The corresponding SEM results are shown in Figure 7. As seen in Figure 7a, when no current was applied (C_24_I_0_P), the adhesive part (soluble silicate) of the carbon paste and conductive graphite particles were bonded firmly and formed a dense structure. A small amount of diffused Cl (0.88%) was detected Figure 7d.

When 5 mA currents were applied, the bonding between adhesive paste and graphite particles appears to be broken and a more porous structure forms (Figure 7b). The pores may have provided more incursion pathway for anions to reach the CFRP anode. The content of Cl increased to 4.21% compared with that of C_24_I_0_P, combining the XRD result, in the form of NaCl crystals.

When 10 mA currents were applied, smooth particles with size of tens of microns and the loose features around the smooth particles were observed as indicated by black arrows Figure 7c. Meanwhile, the content of Cl increased to 59.32% (smooth particle) and 2.66% (loose feature). The loose feature material seems to have a similar composition to the original carbon paste (no current applied), and it might be the remaining unreacted part of the paste. The element Cl might come from the surrounding region since the detecting area of EDS is of microns. The smooth particles were supposed to be the NaCl crystals either from the diffusion of the NaCl solution used or the Chlorination of trona [Na_3_H(CO_3_)_2_•2H_2_O]. Thus the formation and accumulation of NaCl crystals at the contact material/CFRP interface was estimated to be the main reason for the failure of bonding.

**Figure 8:**
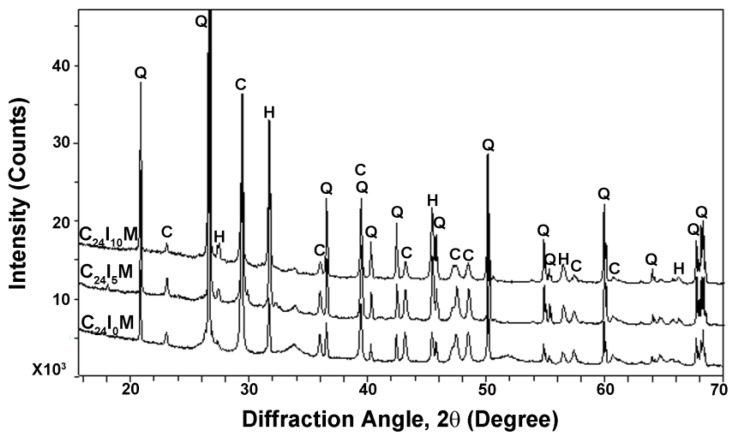
XRD spectra identification of mortar pastes subject to varied current densities. Q-Quartz (SiO_2_), C-Limestone (CaCO_3_), H-Halite (NaCl).

For the mortar contact material, the XRD results are shown in Figure 8. The quartz (SiO_2_) phase was found in all specimens, which is the dominant component of the sand used. Limestone (CaCO_3_) was also detected in all specimens, originating from the carbonation of the hydration product Ca (OH)_2_. In C_24_I_0_M, crystalline halite (NaCl) was detected, which is supposed from the diffusion of NaCl solution. Whereas in C_24_I_5_M and C_24_I_10_M, the content of NaCl increased with current, indicating the applied current accelerated the movement of NaCl from solution to the anode.

**Figure 9:**
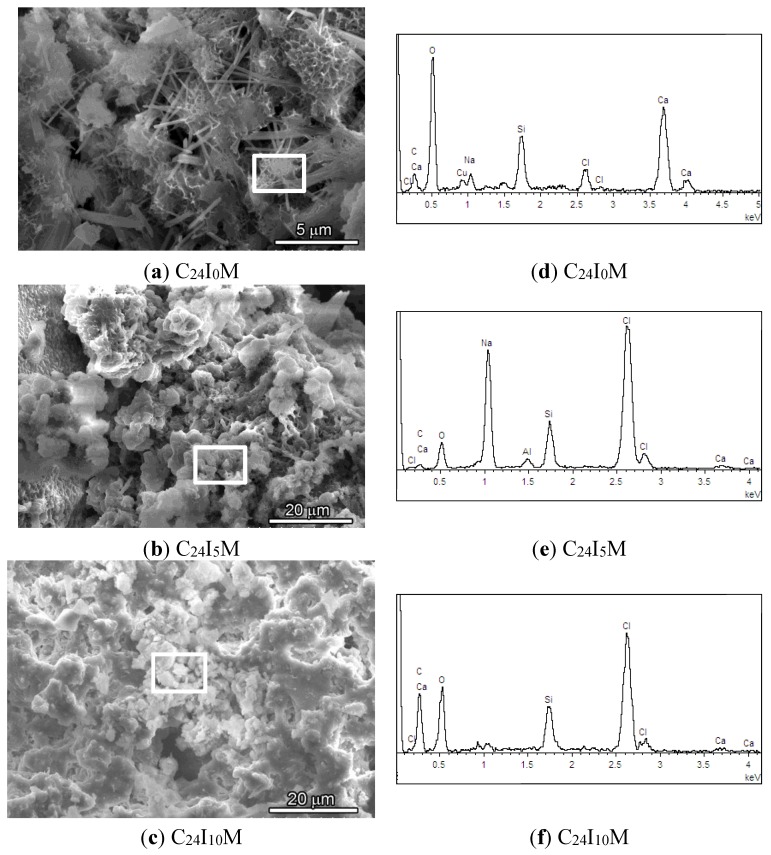
SEM images of the mortar from sample (**a**) C_24_I_0_M; (**b**) C_24_I_5_M and (**c**) C_24_I_10_M. The element analysis results (EDS) of the corresponding area indicated by rectangular frame are shown in (**d**–**f**).

The microstructure and element analyzing results of specimens with mortar contact material are shown in Figure 9. At the same age, mortar with no current applied (C_24_I_0_M) shows a typical fibrous morphology of hydration product, such as C–S–H foil matrix and Ca(OH)_2_ and ettringite fibers (Figure 9a). However, when current was applied, the fibrous structure almost (C_24_I_5_M, Figure 9b) or totally (C_24_I_10_M, Figure 9c) disappeared, replaced by a more dense structure consisting of agglomerated irregular shape particles, which is the later hydration characteristics of cementitious materials. Thus the applied current appears to accelerate the hydration of cementitous materials.

The EDS results show that the content of the element Cl increased from 2.88% to 29.08% for C_24_I_5_M and 11.70% for C_24_I_10_M. Combining with the finding in XRD, the increased content Cl may be from the NaCl accumulation at the anode surface when current applied, which will reduce the bonding area between contact material/CFRP. Moreover, the content of element Ca was measured and found to decrease with increasing current, from 13.57% (C_24_I_0_M) decreased to 0.63% (C_24_I_5_M) and 0.40% (C_24_I_10_M) according to the quantification of EDS results, which suggests the transport of cationic Ca^2+^ to the cathode. It is known that the bonding component in cementitous material is mainly C–S–H, where Ca is the important consisting element. The loss of Ca will cause the degradation of C–S–H and thus the failure of bonding capability, which is consistent with the finding in Ref [23]. Consequently, both the accumulation of NaCl and the loss of Ca at the anode will cause the loss of shear strength of the interface and the increase of electrical resistance over time. Further study of the mechanism will be a focus of future studies.

### 3.5. CFRP Anode

The influence of the polarization process on CFRP was assessed by the corrosion depth, from which the corrosion rate (corrosion depth/duration time) was also estimated. After 25 days of polarization, the corrosion depths of C_24_I_0_P, C_24_I_5_P, and C_24_I_10_P were approximately 0, 0, and 0.019 mm, respectively. The calculated corrosion rate is approximately 0.7 μm/day. In comparison, the corrosion depth of the mortar series was 0, 0.119 mm, and 0.136 mm for C_24_I_0_M, C_24_I_5_M, and C_24_I_10_M, respectively, with a calculated corrosion rate of approximately 5 μm/day. Consequently, the CFRP anode using carbon paste as the contact material can survive an order of magnitude longer than the one using mortar, which may be related to the different pore structures of the contact materials, consistent with the observation by digital camera (Figure 4 and Figure 5). The corrosion mechanism on CFRP has been studied in [24]. It was found that Cl_2_ and more oxidizing HClO will be produced at the anode (CFRP) region during electrolysis when Cl^−^ exists in the electrolyte, which will decompose the epoxy in CFRP into powders and cause a rough surface of CFRP as observed in Figure 4 and Figure 5. We should also note that the failure occurred at the interface between contact material/CFRP instead of concrete/contact material. This indicates that the anode in this polarization system is CFRP instead of contact material since only the electron/ion exchange instead of electron exchange or ion exchange can cause damage. The electron/ion exchange at the interface is a typical feature of anodic reaction.

Additionally, although the CFRP was corroded up to 0.136 mm in depth, the weakest part of the polarization system where fracturing occurred was still at the interface between the CFRP and the contact material rather than inside the CFRP, according to Figure 4 and Figure 5. This indicates that the corrosion of CFRP is not the primary cause of the degradation of the contact material/CFRP interface during 25 days of polarization.

## 4. Conclusions

An experimental investigation was carried out to study the interface deterioration of reinforced concrete specimens with CFRP anode after polarization. Two contact materials of carbon fiber reinforced mortar and conductive carbon paste were used to attach the CFRP anode with concrete. The feeding voltage was monitored during polarization period. The underlying degradation mechanisms at the interface between the CFRP anode and contact materials were revealed by means of SEM and XRD techniques. Based on the results and analyses, the following conclusions can be drawn.

1. The feeding voltage increased even though an effective electrical connection was still maintained over the polarization period. The increase of feeding voltage indicates a deterioration of the electrical connection in the system, since the current remained constant during the test period. The deterioration was found resulting from deterioration of the interface between the CFRP anode and contact material.

2. The deterioration of the interface was considered mainly caused by the chemical change of contact materials. For carbon paste contact material, the formation and accumulation of NaCl crystals at the contact material/CFRP interface was estimated to be the main reason for the failure of bonding. For mortar contact material, both the accumulation of NaCl and the loss of element Ca at the anode are main reasons. The deterioration of the interface led to the loss of shear strength of the interface and the increase of electrical resistance over time.

3. The CFRP with mortar as the contact material was corroded more severely (5 μm/day) than that with carbon paste as the contact material (0.7 μm/day) after 25 days polarization. Thus, from the aspect of durability of CFRP, the carbon paste seems more protective for CFRP than mortar when the electrical connection is ensured.

4. Large current densities were adopted in the present tests to achieve possible interface deterioration of the polarization system in a relatively short period. Further research with practical current densities is necessary before site application to investigate the long-term behavior of reinforced concrete ICCP system with CFRP anode.
